# Elevation of Soluble Guanylate Cyclase Suppresses Proliferation and Survival of Human Breast Cancer Cells

**DOI:** 10.1371/journal.pone.0125518

**Published:** 2015-04-30

**Authors:** Hui-Chin Wen, Chih-Pin Chuu, Chen-Yu Chen, Shine-Gwo Shiah, Hsing-Jien Kung, Kuang-Liang King, Liang-Chen Su, Shi-Chuan Chang, Chung-Ho Chang

**Affiliations:** 1 Institute of Cellular and System Medicine, National Health Research Institutes, Miaoli County, Taiwan; 2 National Institute of Cancer Research, National Health Research Institutes, Miaoli County, Taiwan; 3 Institute of Molecular and Genomic Medicine, National Health Research Institutes, Miaoli County, Taiwan; 4 Division of General Surgery, Department of Surgery, Taipei Veterans General Hospital, Taipei City, Taiwan; 5 Chest Department, Taipei Veterans General Hospital, Taipei City, Taiwan; 6 Institute of Emergency and Critical Care Medicine, National Yang-Ming University, Taipei City, Taiwan; 7 Graduate Institute of Basic Medical Science, Ph.D. Program of Aging, China Medical University, Taichung City, Taiwan; Wayne State University, UNITED STATES

## Abstract

Nitric oxide (NO) is an essential signaling molecule in biological systems. Soluble guanylate cyclase (sGC), composing of α1 and β1 subunit, is the receptor for NO. Using radioimmunoassay, we discovered that activation of sGC by treatment with bradykinin or sodium nitroprusside (SNP) is impaired in MCF-7 and MDA-MB-231 breast cancer cells as compared to normal breast epithelial 184A1 cells. The 184A1 cells expressed both sGC α1 and sGCβ1 mRNAs. However, levels of sGCβ1 mRNAs were relatively lower in MCF-7 cells while both mRNA of sGC subunits were absent in MDA-MB-231 cells. Treatment with DNA methyltransferase inhibitor 5-aza-2’-deoxycytidine (5-aza-dC) increased mRNA levels of both sGCα1 and sGCβ1 in MDA-MB-231 cells but only sGCβ1 mRNAs in MCF-7 cells. The 5-aza-dC treatment increased the SNP-induced cGMP production in MCF-7 and MDA-MB-231, but not in 184A1 cells. Bisulfite sequencing revealed that the promoter of sGCα1 in MDA-MB-231 cells and promoter of sGCβ1 in MCF-7 cells were methylated. Promoter hypermethylation of sGCα1 and sGCβ1 was found in 1 out of 10 breast cancer patients. Over-expression of both sGC subunits in MDA-MB-231 cells induced apoptosis and growth inhibition *in vitro* as well as reduced tumor incidence and tumor growth rate of MDA-MB-231 xenografts in nude mice. Elevation of sGC reduced protein abundance of Bcl-2, Bcl-xL, Cdc2, Cdc25A, Cyclin B1, Cyclin D1, Cdk6, c-Myc, and Skp2 while increased protein expression of p53. Our study demonstrated that down-regulation of sGC, partially due to promoter methylation, provides growth and survival advantage in human breast cancer cells.

## Introduction

Nitric oxide (NO) is an essential signaling molecule in biological systems. NO functions as the primary activator of soluble guanylate cyclase (sGC) [1]. NO is synthesized by the enzyme nitric oxide synthase (NOS) [1]. Once synthesized, NO diffuses across cell membranes and binds to the heme cofactor of sGC and activates the enzyme, which leads to significant increases in cGMP levels [1–4]. NO donors can also efficiently activate sGC [5–8]. The second messenger, cGMP, then directly modulates ion channels, cGMP-phosphodiesterases, or cGMP-dependent protein kinases (PKG) and therefore regulates important physiological functions, including vasodilation, platelet aggregation, and neurotransmission [1].

NO is reported to be harmful for adipogenic milieu of the breast, where NO initiates and promotes tumorigenesis [9]. Epidemiological studies revealed that people with higher risks for developing estrogen receptor (ER)-positive breast cancer express specific polymorphic forms of endothelial NOS which continuously produce sustained low levels of NO [10]. The NO then generates oxidative stress and inflammatory factors and alters the microenvironment of the breast, providing an environment for the transformation of breast cancer cells [10]. NOS activity has been reported to be higher in the advanced breast tumors than that in normal or benign breast tissues [11, 12]. Patients with triple-negative breast tumors expressing NOS have significantly worse prognosis [13]. However, the precise mechanism by which the NO-sGC signaling modulates proliferation of breast cancer cells is not clear. Previously, we found that the bradykinin/sGC signaling pathway is functional in androgen-dependent LNCaP prostate cancer cells, but is impaired in androgen-independent PC-3 and DU 145 prostate cancer cells [14]. Neither NO activator bradykinin nor NO-donor sodium nitroprusside (SNP) can activate the sGC in PC-3 and DU 145 cells [14]. Similar findings have later been reported by other groups [15, 16]. In this study, we investigated if the NO-sGC signaling is impaired in the human breast cancer cells and also determined the effect of sGC expression on cell proliferation and survival both *in vitro* and *in vivo*.

## Materials and Methods

### Tissue samples, cell lines, materials

Breast tumors and adjacent tissue were obtained from patients in Taipei Veterans General Hospital (Taipei city, Taiwan). The Institutional Review Board (IRB) of Taipei Veterans General Hospital approved the study, and written informed consent was acquired from the patients following to the ethical guideline from IRB (VGH-IRB No.: 201010001IA). Human breast cell line 184A1 (kindly provided from Dr. Hung, MC in Center of Molecular Medicine and Graduate Institute of Cancer Biology, China Medical University & Hospital, Taiwan) were cultured in DMEM/F12 supplemented with 5% (v/v) horse serum, 100 U/mL penicillin, 100 μg/mL streptomycin, 20 ng/mL hEGF, 10 μg/mL insulin and 500 ng/mL hydrocortisone. Breast cancer cell lines MCF-7 (kindly provided from Dr. Yu, WCY in National Institute of Cancer Research, National Health Research Institutes, Taiwan) and MDA-MB-231 (purchased from Bioresource Collection and Research Center, Taiwan) were grown in PRMI1640 medium supplemented with 10% fetal bovine serum, 100 U/mL penicillin and 100 μg/mL streptomycin. All cell lines were maintained at 37°C in a 5% CO_2_ humidified atmosphere. The doubling time of 184A1, MCF-7, and MDA-MB-231 cells used in our experiments was 41.6, 38.4, and 39.7 h, respectively. Bradykinins, 5-Aza-2’-deoxycytidine (5-aza-dC), SNP, MTT and other chemicals were purchased from Sigma (St. Louis, MO, USA).

### Cyclic GMP determination

184A1, MCF-7 and MDA-MB-231 cell lines were grown to confluence in 6-well plates. The cells were washed with 2 mL of serum-free medium (pH7.3), and then incubated with 900 μL of medium containing 0.5 mM isobutylmethylxanthine at 37°C for 10 min. 10 nM bradykinin or 50 μM SNP was added to the cells and incubated at 37°C for an additional 10 min. After incubation, the medium was aspirated and 1 mL cold 10% trichloroacetic acid was added to the plates. The cell extracts were scraped and centrifuged for 15 min at 2000 x *g*. The supernatant fractions were then extracted with water-saturated ether to remove trichloroacetic acid. The cGMP level in the supernatants was determined by radioimmunoassay [17].

### Nucleic acid extraction

DNA from cell lines was extracted using PureLink Genomic DNA Purification Kit (Invitrogen, Carlsbad, CA, USA) according to the manufacturer’s instructions. Total RNA from cell lines was extracted using TRIZOL Reagent (Invitrogen). DNA and total RNA from tissue were homogenized by TissueLyser II (Qiagen, Hilden, Germany) according to the manufacturer’s instructions and extracted using TRIZOL Reagent.

### Quantitative reverse transcriptase polymerase chain reaction (qRT-PCR)

Total RNA (2–4 μg) was reverse transcribed using RevertAid H Minus First Strand cDNA Synthesis Kit (Fermentas/Thermo Fisher Scientific). Quantification real-time PCR was using QuantiNova SYBR Green PCR Kit (Qiagen) and performed on Applied Biosystems 7500 Real-Time PCR System. Sequences of the qPCR primer pairs were as follows: sGCα1-F, 5’-AAATCAATGTCAGCCCAACA-3’; sGCα1-R, 5’-AAACACGAAACCAGGACAGTC-3’; sGCβ1-F, 5’-GCCAGGTTCAAGTAGATGGTG-3’; sGCβ1-R, 5’-GGCATCCGCTGTCCTATG-3’ [18]; PUMA-F, 5’-GACGACCTCAACGCACAGTA-3’; PUMA-R, 5’-AGGAGTCCCATGATGAGATTGT-3’ [19]; PBGD-F, 5’-AGTGTGGTGGGAACCAGC-3’; PBGD-R, 5’-CAGGATGATGGCACTGAACTC-3’ [20]; ACTB-F, 5’-GGAAATCGTGCGTGACATT-3’; ACTB-R, 5’-GGAGTTGAAGGTAGTTTCGTG-3’. The relative mRNA levels from cell lines were calculated using the 2^-ΔΔ*C*T^ method [21], with ACTB expression as a normalizer. And those from clinical tissue were normalized to the level of PBGD expression. The clinical relative sGCα1 and sGCβ1 mRNA ratio (Tumor/Normal) was determined as follows:

$$R=\frac{sGCtumor/PBGDtumor}{sGCnormal/PBGDnormal}$$

### Bisulfite sequencing

Bisulfite modification of genomic DNA was carried out by EZ DNA Methylation-Gold Kit (Zymo Research, Irvine, CA, USA). Modified DNA was amplified by PCR with following primers: 5’-GTAAGGAGGATTGTTTGGGAGTTA-3’ (forward) and 5’- AAAAAACCCTACAATAACACCTATC-3’ (reverse) for sGC α1 CpG island; 5’-TTTTGTTTTTGATTGGTTGAAGAA-3’ (forward) and 5’-AAACACTTACCATAATATCTACACC-3’ (reverse) for sGC β1 CpG island. PCR was performed by Maxima Hot Start *Taq* DNA Polymerase (Fermentas/Thermo Fisher Scientific), initiated by denaturing at 95°C for 5 min, followed by 40 cycles of 95°C for 30 sec, 55°C for 30 sec, 72°C for 30 sec, and a final extension step at 72°C for 5 min. The amplicon was cloned into pGEM-T Vector System (Promega, Fitchburg, Wisconsin, USA), with 6 to 8 individual colonies were randomly chosen and sequenced by the DNA sequencing core facility at National Health Research Institutes (Taiwan).

### Antibodies

Dnmt1, Dnmt3a, Cdc25A and GAPDH antibodies were purchased from GeneTex (Hsinchu city, Taiwan). Bcl-2, Bcl-xL, Cdk6, Dnmt3b, and p53 antibodies were purchased from Cell Signaling Technology (Danvers, MA, USA). The c-Myc, sGCα1 and CyclinD1 antibodies were purchased from Epitomics/Abcam (Cambridge, UK). The sGCβ1 antibody was purchased from Calbiochem/Millipore (Billerica, MA, USA). Cyclin B1 antibody was purchased from Upstate Biotechnology/Millipore (Lake Placid, NY, USA). Cdc2 and Skp2 antibody were purchased from Santa Cruz (Dallas, TX, USA).

### Western blotting

Cell lines were washed by PBS and lysed with 1% Triton X-100 in 20 mM Sodium phosphate buffer (pH7.4) containing 150 mM NaCl, supplemented with Protease Inhibitor Cocktail (#11873580001) from Roche (Basel, Switzerland), 14 μg/mL aprotinin and 10 μg/mL leupeptin. The extracted protein concentrations were determined by the BCA protein assay (Pierce/Thermo Scientific; Waltham, MA, USA). Equal amount of protein were separated on SDS-PAGE and transferred to PVDF membrane (Millipore). The membrane was incubated with specific antibodies, detected with HRP-conjugated secondary antibodies and visualized by Western Lightning Plus-ECL, Enhanced Chemiluminescence Substrate (PerkinElmer, Waltham, MA, USA) [17, 22, 23].

### Expression constructs

The Open-Reading-Frames of sGCα1 and sGCβ1 were PCR-amplified form cDNA of 184A1 cells by *Pfu* DNA Polymerase (Fermentas/Thermo Fisher Scientific) and cloned into pcDNA3.1/Zeo(+) (Invitrogen) and pcDNA3.1(-) (Invitrogen), respectively. Plasmid DNAs were purified and sequenced by the DNA sequencing core facility at National Health Research Institutes (Taiwan).

### Stable transfection

MDA-MB-231 cells were grown in 6-well plates for 24 hr and transfected with plasmids containing sGCα1 and sGCβ1 using Lipofectamine 2000 (Invitrogen) according to the manufacturer’s instructions. After 24 hr, fresh medium containing 500 μg/mL G418 and 250 μg/mL Zeocin (Invitrogen) was added for selection. After 3 weeks, individual colonies of stably-transfected cells were picked and maintained in medium containing 400 μg/mL G418 and 100 μg/mL Zeocin.

### Cell proliferation assay

Cell proliferation was determined by the MTT assay as previously described [24]. Briefly, cells were plated into 96-well plates at a density of 2.5 x 10^3^ cells per well and measured every 24 hr. MTT assay was performed by adding 5 mg/mL MTT solution to each culture to equal one tenth the original culture volume and incubated at 37°C for 2 hr. The medium was removed and 50 μL DMSO was added to each culture at room temperature for 20 min with gentle shaking. Absorbance of converted dye is measured at a wavelength of 570 nm with background subtraction at 650 nm.

### Cell cycle analysis

Cell cycle distribution was determined by flow cytometric analysis as previously described [25]. 5 x 10^5^ cells were harvested, fixed in 50% ice-cold ethanol and stored at 4°C for later analysis. The fixed cells were centrifuged at 200 x *g* for 7 min, resuspended in 0.5 mL PBS containing 50 μg/mL propidium iodide and 200 μg/mL RNase and incubated for 20 min at room temperature in the dark. The Cells were analyzed with a BD FACSCalibur flow cytometer (BD Bioscience) equipped with BD CellQuest Pro software. ModFit LT v.3 software was used to analyze the percentage of cells in different phases of the cell cycle.

### Caspase 3 activity assay

Cells were harvested in cell lysis buffer containing 50 mM PIPES (pH7.2), 50 mM KCl, 5 mM EGTA, 2 mM MgCl_2_, frozen and thawed twice, and centrifuged at 12000 x *g* for 3 min. 40 μg of soluble cell lysate in 100 μL cell lysis buffer was added to 100 μL reaction buffer containing 40 mM HEPES (pH7.2), 20% glycerol, 200 mM NaCl, 10 mM DTT, and 10 mM phenylmethylsulfonyl fluoride, to achieve a total reaction volume of 200 μL. The reaction was initiated by 40 μM Ac-DEVD-AFC (Enzo Life Sciences, Farmingdale, NY) as final concentration and incubated at 37°C for 1 hr in the dark. Caspase 3 activity was assessed by measuring fluorescence at excitation wavelength of 400 nm and emission wavelength of 505 nm using EnSpire 2300 Multilabel Reader (PerkinElmer).

### Apoptosis assay

Apoptosis assay was performed by ApoStrand ELISA Apoptosis Detection Kit (Enzo Life Sciences) according to the manufacturer’s instructions.

### Tumor Xenografts in Athymic Mice

Experiments involving mice were approved by the Institutional Animal Care and Use Committee at National Health Research Institutes following the guidelines for the care and use of animals that cover the research and were adhered to the ARRIVE guidelines. Female Balb/c nu/nu mice (National Laboratory Animal Center, Taiwan), 8 weeks of age, were injected subcutaneously in both flanks with 5 x 10^5^ MD-MBA-231 breast cancer stable cells over-expressing sGC or MD-MBA-231 cells with control vector. MD-MBA-231 breast cancer cells were suspended in DMEM medium without FBS and mixed with equal volume of Matrigel (BD Bioscience, Franklin Lakes, NJ, U.S.A.). Cells were confirmed for mycoplasma free by using PCR for medium detection before every experiment. Tumors growth and body weight were monitored weekly one week after tumor inoculation. Mice were separated into control and treatment groups. There were 4 mice in control group and 5 mice in sGC over-expression group. Tumors were measured weekly using calipers. Tumor volume was calculated using the formula volume = length x width x height x 0.52 [24–26]. No significant difference of body weight was observed between mice in control group and mice in sGC over-expression group. End point was defined as 16^th^ weeks after cancer cell injection or if mice show syndrome meet criteria for early removal, including 1. Body weight reduction over 20% 2. Inability to intake food or water 3. Inability to move 4. Back arching 5. Any other physical syndromes showed that the mice are severe sick. Mice control 1 was sacrificed on 8^th^ week due to over-sized tumors. All other mice were sacrificed on 16^th^ week. Mice were euthanized after completion of the experimental protocol, at development of specific signs, when weight loss of 20% occurs, or when moribund. The sacrifice of mice was via euthanasia using 100% CO_2_ exposure for 5–10 min via inhalation. Heart beeping was checked after the euthanasia to ensure that the mice were dead.

## Results

### The NO-sGC-cGMP signaling pathway is impaired in human breast cancer MCF-7 and MDA-MB-231 cells

**Fig 1:**
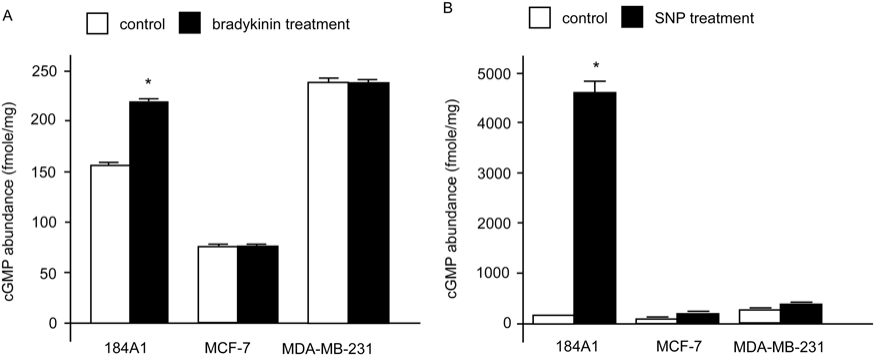
Effects of bradykinin and SNP on cGMP formation in human breast epithelial 184A1 cells and human breast cancer MCF-7 and MDA-MB-231 cells. Bradykinin (A) or SNP (B) induced cGMP was measured by radioimmunoassay. The error bar represents the standard deviation from the mean of the four replicates. Asterisk * indicated statistically significant difference of *p* value < 0.05 between the two groups being compared.

To examine whether the NO synthase-sGC signaling pathway is functional in human breast cancer cells, we measured the effects of bradykinin, a NO activator, on the production of cGMP in human 184A1 breast epithelial cells, human ER-positive MCF-7 breast cancer cells, and ER-negative MDA-MB-231 breast cancer cells. Bradykinin treatment at 10 nM caused 55% increase of sGC in 184A1 cells, but not in MCF-7 and MDA-MB-231 cells (Fig 1A). To determine whether sGC functions properly, we measured the effect of SNP, a NO donor and sGC activator, on sGC activity in 184A1, MCF-7 and MDA-MB-231 cells. SNP treatment (50 μM) effectively stimulated sGC activity in 184A1 cells but not in MCF-7 and MDA-MB-231 cells (Fig 1B). These results suggested that the NO-sGC-cGMP signaling pathway is impaired in MCF-7 and MDA-MB-231 human breast cancer cells.

### The impaired NO-sGC-cGMP signaling in breast cancer cells is due to reduced expression of sGC

**Fig 2:**
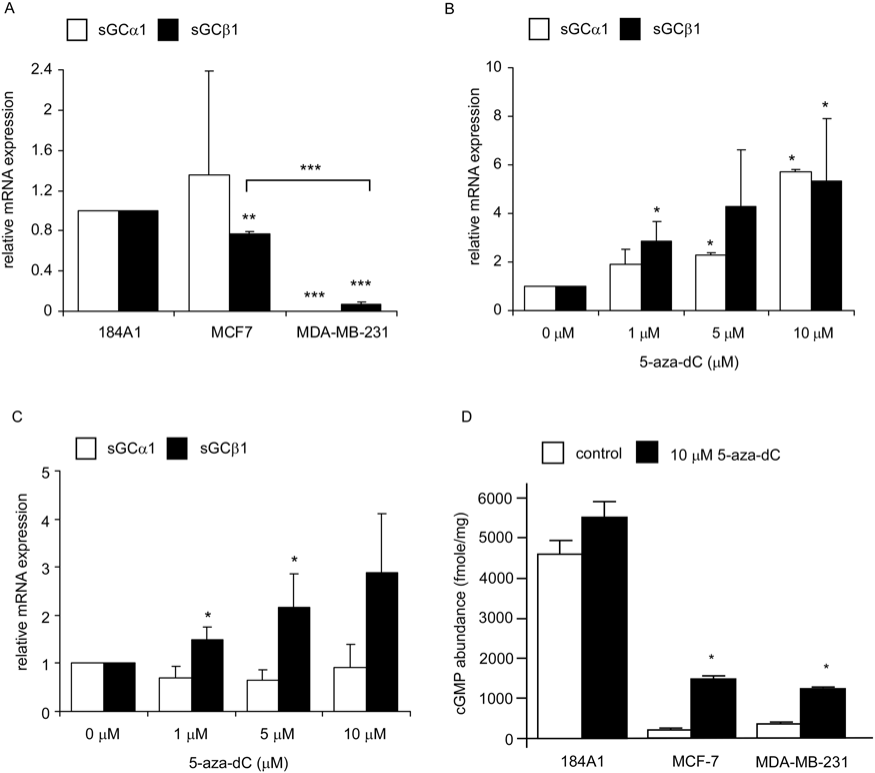
Expression of sGC subunits in human breast epithelial and cancer cell lines. (A) Expression of sGCα1 and sGCβ1 mRNA were determined by qRT-PCR in 184A1, MCF-7 and MDA-MB-231 cells. Relative level was normalized by ACTB. Effects of 5-aza-dC treatment on the expression of sGCα1 and sGCβ1 mRNA in MDA-MB-231 (B) and MCF-7 (C) cells were analyzed by qRT-PCR. (D) Effect of 5-aza-dC on activation of sGCα1 and sGCβ1 was assayed by radioimmunoassay for cGMP production. The error bar represents the standard deviation from the mean of the four replicates. Asterisk * and *** indicated statistically significant difference of *p* value< 0.05 and *p* value< 0.001, respectively, between the two groups being compared.

The sGC is a heterodimer, composing of α and β subunit. We used qRT-PCR to determine whether low expression level of sGC subunits is responsible for the impaired sGC activation in human breast cancer cells. Unlike 184A1 cells, the mRNA of both sGC α 1 and sGC β 1 were extremely low in MDA-MB-231 cells (Fig 2A), which may account for its inability to be activated by bradykinin or SNP. On the other hand, although the mRNA expression levels of sGC α 1 subunit were not very different in 184A1 and MCF-7 cells, the mRNA expression levels of sGCβ1 in MCF-7 cells were less than those in 184A1 cells (Fig 2A).

### Promoter methylation contributed to the reduced expression of sGC in breast cancer cells

Promoter methylation is a common mechanism to abolish gene expression. We examined whether reduced expression of sGCα1 and sGCβ1 mRNA in breast cancer cells is due to promoter methylation. Expression of sGCα1 and sGCβ1 mRNA in MDA-MB-231 cells (Fig 2B) as well as sGCβ1 mRNA in MCF-7 cells (Fig 2C) were significantly increased by treatment of DNA methylation inhibitor 5-aza-dC. These results suggested that DNA methylation contributes, at least partially, to the low expression of sGCα1 and sGCβ1 in breast cancer cells. To examine whether sGC is functionally expressed in MCF-7 and MDA-MB-231 cells after 5-aza-dC treatment, we pretreated 184A1, MCF-7 and MDA-MB-231 cells with 5-aza-dC before challenging cells with SNP. Activity of sGC was significantly enhanced in both MCF-7 and MDA-MB-231 cells after 5-aza-dC treatment (Fig 2D). In contrast, the activity of sGC in 184A1 cells was not affected by 5-aza-dC treatment (Fig 2D).

### Promoter methylation of sGCα1 and sGCβ1 in breast cancer cells

**Fig 3:**
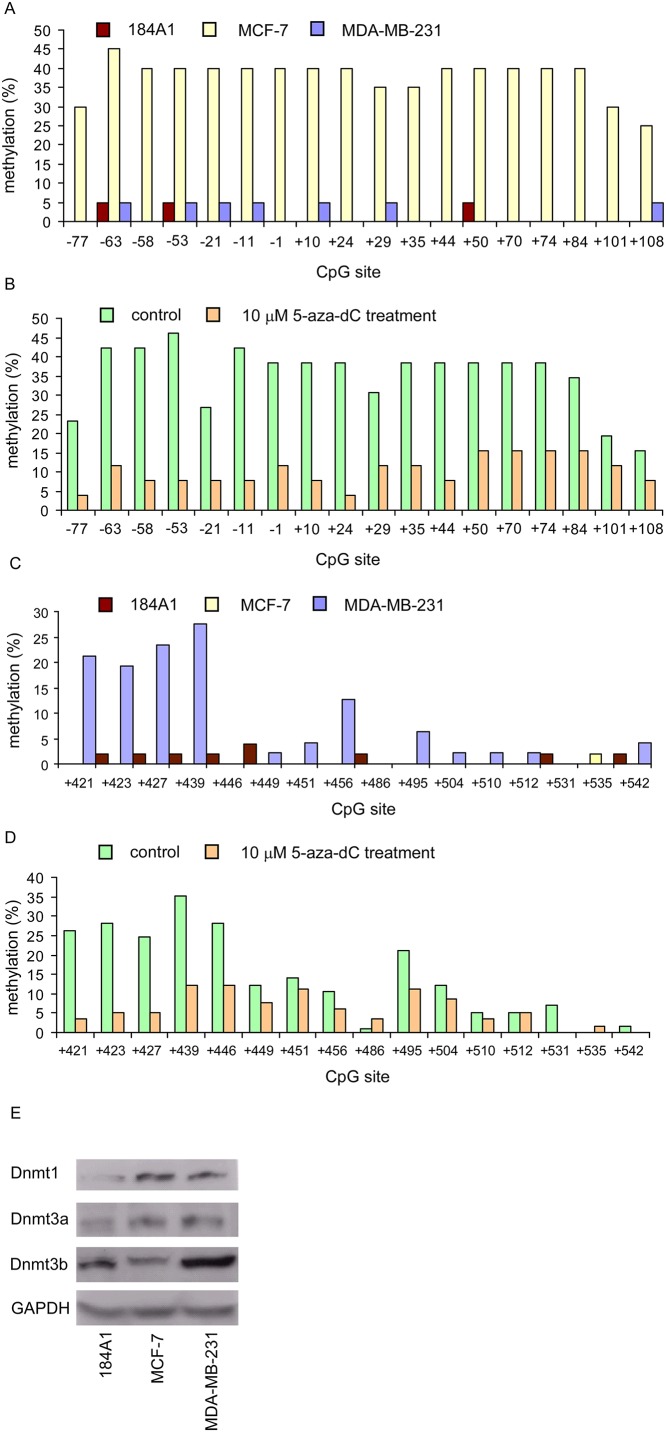
Promoter hypermethylation of sGCβ1 in MCF-7 and sGCα1 in MDA-MB-231 cells. Promoter methylation of sGCβ1 (A) and sGCα1 (C) in 184A1, MCF-7, and MDA-MB-231 cells was analyzed by bisulfite sequencing. Effects of 5-aza-dC treatment (10 μM, 72 hr) on status of promoter methylation of sGCβ1 (B) in MCF-7 cells and sGCα1 (D) in MDA-MB-231 cells were analyzed by bisulfite sequencing. The y-axis represents the percentage of methylation at each CpG site; methylation percentage of each individual CpG site was determined by dividing the number of methylated cytosine by the total number of colonies (methylated plus unmethylated) analyzed. The x-axis represents the nucleotide positions relative to the sGCβ1 or sGCα1 transcription start sites. (E) Protein levels of the endogenous DNA methltransferases (Dnmts) in MCF-7 and MDA-MB-231 cells were compared with 184A1 cells by Western blotting. GAPDH protein expression was used as loading control.

To determine whether promoter regions of sGCα1 and sGCβ1 are indeed methylated, we used MethPrimer software to identify putative CpG islands. We identified two putative CpG islands in the 5’ flanking region of sGCα1 and sGCβ1. One resides in the sequences between nt 81135820 and nt 81136367, the other locates in the sequences between nt 81227831 and nt 81227969 (GenBank accession no. NT_016354). We thus used bisulfite sequencing to determine whether CpG islands of sGCα1 and sGCβ1 are methylated in MCF-7 and MDA-MB-231 cells. As shown in Fig 3A, the promoter region of sGCβ1 in MCF-7 was highly methylated. The methylation of these promoters can be reduced by 5-aza-dC treatment (Fig 3B). On the other hand, promoter region of sGCα1 in MCF-7 cells was not methylated (Fig 3C). In MDA-MB-231 cells, several CpG islands in the 5’ flanking region of sGCα1 were observed methylated (Fig 3C). The methylation of these promoters can be attenuated by 5-aza-dC treatment (Fig 3D). Surprisingly, we did not detect much methylation of sGCβ1 in MDA-MB-231 cells (Fig 3A). In contrast to MCF-7 and MDA-MB-231 cells, very little methylation of sGCα1 and sGCβ1 promoter was detected in 184A1 cells (Fig 3A and 3C). DNA methylation is catalyzed by DNA methyltransferases (Dnmts), including Dnmt1, Dnmt2 (TRDMT1), Dnmt3a, and Dnmt3b. Among these enzymes, over-expression of Dnmt3b have been found in the tumor tissues of approximately 30% of breast cancer patients, whereas Dnmt1 and Dnmt3b are over-expressed in approximately 5% and 3% of breast tumors [27]. We compared protein levels of Dnmt1, Dnmt3a and Dnmt3b in 184A1, MCF-7 and MDA-MB-231 cells. Dnmt1, Dnmt3a and Dnmt3b levels in MDA-MB-231 cells as well as Dnmt1 and Dnmt3a levels in MCF-7 cells were elevated as compared to those in 184A1 cells (Fig 3E). Elevated expression of Dnmts may contribute to the methylation of the promoter of sGC subunits in MCF-7 and MDA-MB-231 cells.

### Promoter methylation of sGCα1 and sGCβ1 in human breast tumors

**Fig 4:**
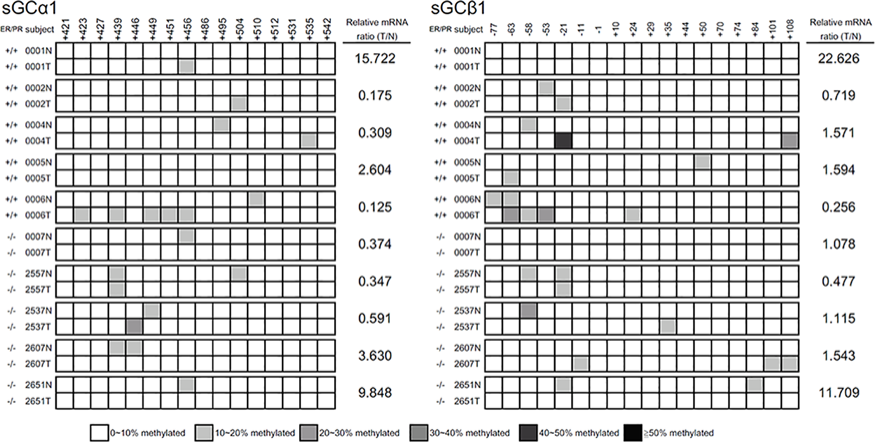
Promoter methylation and mRNA expression of sGCα1 and sGCβ1 in ER+/PR+ and ER-/PR- human breast tumor and normal tissues. Each row of squares represents the cytosine methylation patterns of sGCα1 and sGCβ1 CpG islands, which amplified from individual patient specimens (N: normal tissue; T: tumor tissue). Methylation density is shown at the bottom of the Fig. mRNA expression of sGCα1 and sGCβ1 were determined by qRT-PCR. Relative ratio of sGCα1 and sGCβ1 mRNA expression was normalized to the PBGD gene and its normal tissues.

To examine whether the promoter of sGC subunits is methylated in breast cancer patients, we isolated genomic DNAs from normal and tumor portions of breast tissues from 10 breast cancer patients [5 patients carried ER+/Progesterone Receptor (PR)+ breast tumors, and the other 5 patients carried ER-/PR- breast tumors] (Fig 4). To determine whether the promoter methylation of sGC leads to reduced mRNAs in breast tumors, we performed qRT-PCR analysis to compare sGC mRNA levels in these patients. sGCα1 were down-regulated in 6 patients (No.0002, 0004, 0006, 0007, 2557 and 2537) compared to it’s own normal portions. sGCβ1 were down-regulated in 3 patients (No.0002, 0006, 2557) compared to it’s own normal portions. Among these patients, only patient No.0006 acquired promoter hypermethylation on both sGCα1 and sGCβ1. These results suggested that promoter hypermethylation of sGC subunit may occur in some breast cancer patients.

### Over-expression of sGCα1 and sGCβ1 in MDA-MB-231 cells suppresses cell proliferation, induced apoptosis, and disturbed cell cycle progression

**Fig 5:**
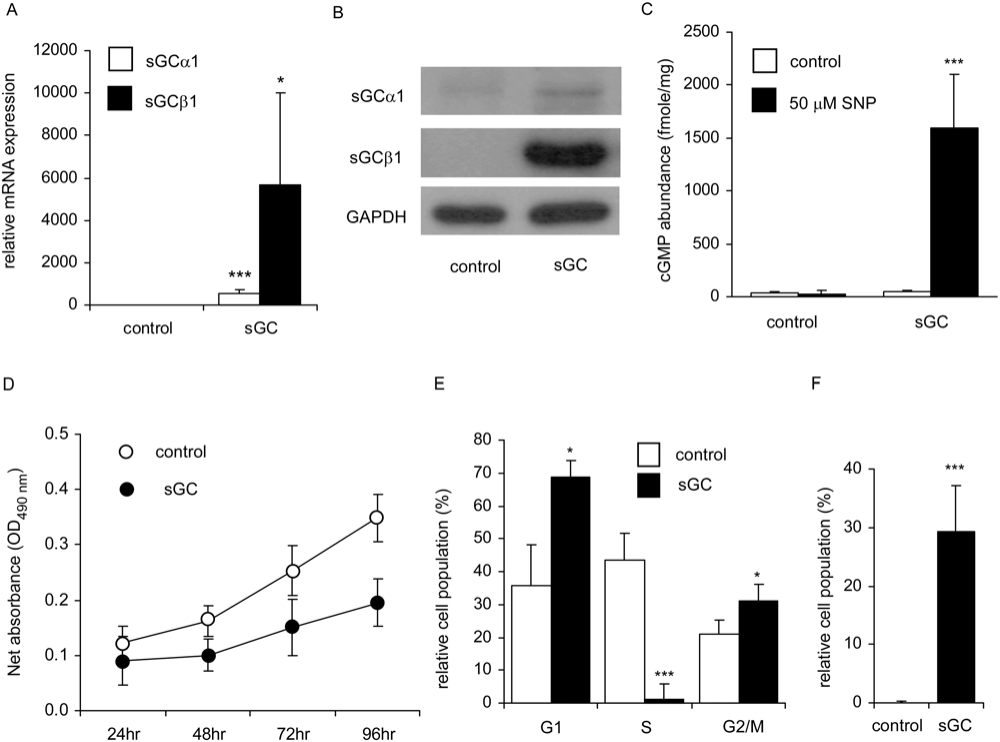
Over-expression of sGCα1 and sGCβ1 in MDA-MB-231 cells affected cGMP production, proliferation, survival, and cell cycle. Normalized level of mRNA (A) and expression level of protein (B) of sGCα1 and sGCβ1 in MDA-MB-231-sGC cells were compared to control cells. (C) SNP-induced cGMP production was compared between MDA-MB-231-control and MDA-MB-231-sGC cells. Results were obtained from 3 independent experiments. Error bars represent mean with standard deviation. Asterisk * and *** indicated statistically significant difference of *p* value< 0.05 and *p* value< 0.001, respectively, between the two groups being compared. (D) Cell proliferation was compared between MDA-MB-231-control and MDA-MB-231-sGC cells after various time periods (24, 48, 72, and 96 hr) by MTT assay. Results were obtained from 3 independent experiments. The error bar represents the standard deviation of the mean. Cell cycle distribution of G1, S, and G2/M (E) or sub-G1 (F) in MDA-MB-231-control and MDA-MB-231-sGC cells was determined by flow cytometric analysis with PI staining. Asterisk * and triple asterisks *** indicated statistically significant difference of *p* value < 0.05 and *p* value < 0.001, respectively, between the two groups being compared.

NO and sGC activators have been shown to cause growth inhibition and induce apoptosis in several cancer cell lines including breast cancer SK-Br-3 and MDA-MB-468 cells [15]. MDA-MB-231 cells express extremely low levels of both sGC subunits (Fig 2A). We examined whether over-expressing sGC may affect proliferation of MDA-MB-231 cells. We generated MDA-MB-231 stable cell lines over-expressing both sGCα1 and sGCβ1 (MDA-MB-231-sGC cells) (Fig 5A and 5B) so that downstream cGMP can be activated. Unlike MDA-MB-231 vector control (MDA-MB-231-control) cells, 50 μM SNP treatment effectively stimulated cGMP formation in MDA-MB-231-sGC cells (Fig 5C). These results indicated that functional sGC is re-expressed in MDA-MB-231-sGC cells.

The MTT assay showed that over-expression of sGC reduced number of viable cells in MDA-MB-231-sGC cells as compared to control cells (Fig 5D). We suspected that cell cycle progression might be affected by the over-expression of sGC. Indeed, S phase population was significantly lower while G1 and G2 population was significantly higher in MDA-MB-231-sGC cells as compared to those in control cells, suggesting the possibility that sGC re-expression induced G1 and G2 cell cycle arrest in these cells (Fig 5E). The sub-G1 population was much higher in MDA-MB-231-sGC cells than that in control cells, indicating that apoptosis may happen in MDA-MB-231-sGC cells (Fig 5F).

**Fig 6:**
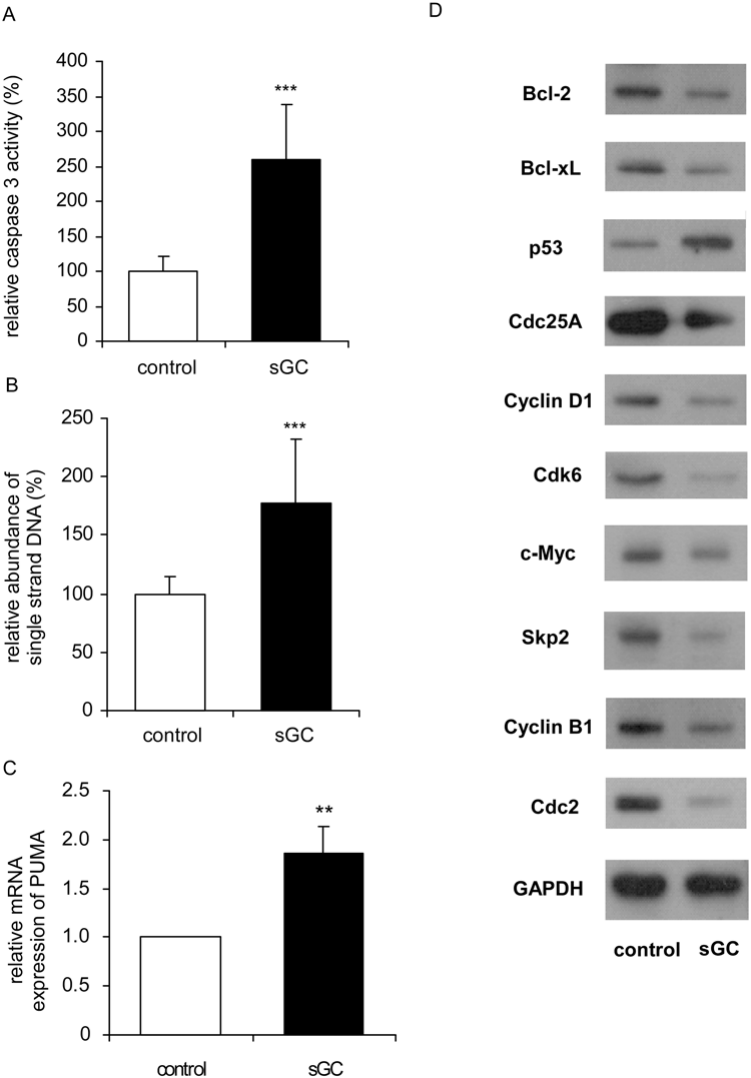
Over-expression of sGC induced apoptosis and cell cycle arrest. (A) Caspase 3 activity and (B) single-strand DNA detection were examined in MDA-MB-231-control and MDA-MB-231-sGC cells for the cell apoptosis. Results were obtained from 3 and 8 independent experiments, respectively. The error bar represents the standard deviation of the mean. (C) Normalized mRNA levels of PUMA in MDA-MB-231-sGC cells were compared to control cells. Asterisk triple asterisks ** and *** indicated statistically significant difference of *p* value < 0.01 and *p* value < 0.001, respectively, between the two groups being compared. (D) Bcl-2, Bcl-xL, p53, Cdc25A, Cyclin D1, Cdk6, c-Myc, Skp2, Cyclin B1, Cdc2 and GAPDH (loading control) proteins in MDA-MB-231-sGC cells were compared to control cells.

To determine whether apoptosis actually happen in MDA-MB-231-sGC cells, we measured the caspase 3 activity in MDA-MB-231-sGC and MDA-MB-231-control cells. Over-expression of sGC subunits caused a 260% induction in caspase-3 activity (Fig 6A). Abundance of formamide-denatured single-stranded DNA (ssDNA), another marker of cell apoptosis, increased significantly in MDA-MB-231-sGC cells as compared to that in control cells (Fig 6B). The mRNA expression of BH3-only protein PUMA (p53 up-regulated modulator of apoptosis) increased dramatically in MDA-MB-231-sGC cells (Fig 6C). Additionally, the protein expression levels of pro-survival Bcl-2 and Bcl-xL decreased while p53 protein levels increased in MDA-MB-231-sGC cells (Fig 6D). Cdc25A, Cyclin D1, Cdk6, c-Myc, and Skp2 (S-phase kinase-associated protein 2) proteins are responsible for G1/S cell cycle check point and progression, while Cyclin B1, c-Myc, and Cdc2 (Cdk1) are responsible for G2/M progression and DNA damage check point. These proteins were significantly down-regulated in MDA-MB-231-sGC cells as compared to control cells (Fig 6D). Taken together, these results suggested that over-expression of sGC subunits induced apoptosis and cell cycle arrest in MDA-MB-231-sGC cells, which in turn reduced the viability of MDA-MB-231-sGC cells.

### Over-expression of sGCα1 and sGCβ1 reduced tumor incidence, tumor volume, and rate of tumor growth of MDA-MB-231-sGC xenografts in nude mice

**Fig 7:**
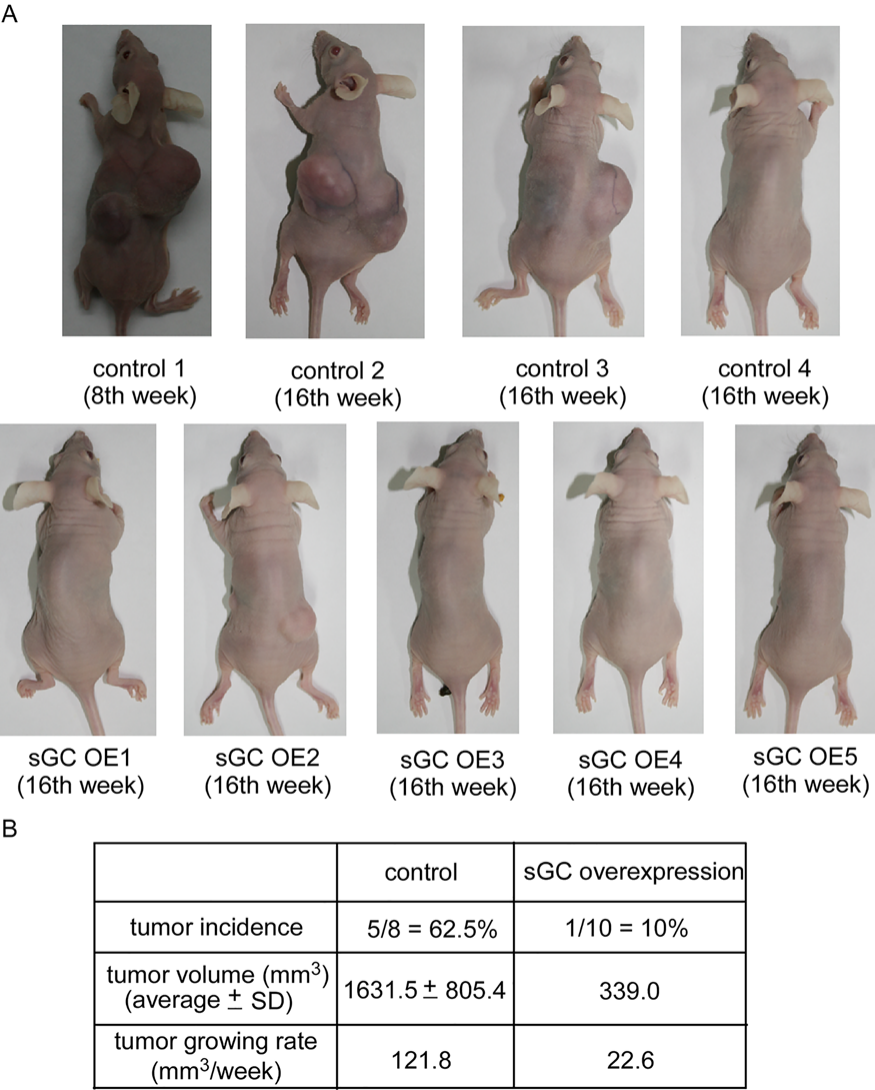
Over-expression of sGC suppressed growth of MDA-MB-231 xenografts in nude mice. Female Balb/c nu/nu mice, 8 weeks of age, were being injected subcutaneously in both flanks with 5 x 10^5^ MDA-MB-231-control or MDA-MB-231-sGC cells suspended in 0.5 ml of Matrigel. Tumors were measured weekly using calipers. Tumor volume was calculated using the formula volume = length x width x height x 0.52. Monitoring of tumors growth was started one week after tumor inoculation. Mice were separated into control and sGC groups. There were 4 mice carrying MDA-MB-231-control xenografts and 5 mice carrying MDA-MB-231-sGC xenografts. Images of all mice before sacrifice were shown in (A). Summary of tumor incidence, average tumor volumes at the end point, and tumor growing rate was listed in (B). End-point tumor volume was shown as mean ± standard error. Tumor growing rate was calculated as summation of tumor volume divided by number of weeks the tumors have grown before mice were sacrificed. Mice control 1 was sacrificed on 8^th^ week due to over-sized tumors. All other mice were sacrificed on 16^th^ week.

Finally, we determined whether over-expression of sGCα1 and sGCβ1 affects cell growth and survival of MDA-MB-231 cells *in vivo*. Four nude mice in control group were injected with MDA-MB-231-control cells in both franks, while five nude mice in sGC group were injected with MDA-MB-231-sGC cells in both franks. Five tumors of MDA-MB-231 successfully developed (62.5%), while only one tumor of MDA-MB-231-sGC developed (10%) (Fig 7A and 7B). Compared to the MDA-MB-231-control tumors, the MDA-MB-231-sGC tumor grew much slower and had a relatively smaller tumor volume (Fig 7B). These results suggested that over-expression of both sGC units reduced the tumor incidence, tumor volume, and tumor growth rate of MDA-MB-231 cells *in vivo*.

## Discussion

In this study, we examined the activation of sGC by bradykinin or SNP in 184A1 breast epithelial cells, MCF-7 and MDA-MB-231 human breast cancer cells. Bradykinin is known to activate sGC through a pathway involving its receptor, Gqα, phospholipase C (β or γ), and NO synthase [28–32]. We found that 10 nM bradykinin increased sGC activity about 55% in 184A1 cells, but had no effect on the enzyme activity in MCF-7 and MDA-MB-231 cells, suggesting that the signaling from the bradykinin receptor to sGC is impaired in MCF-7 and MDA-MB-231 cells. To determine whether defective or inappropriate expression of sGC is responsible for the impaired sGC activation by bradykinin, we stimulated 184A1, MCF-7 and MDA-MB-231 cells with SNP. SNP effectively stimulated sGC activity in 184A1 cells, but had much smaller effects on the enzyme activity in MCF-7 and MDA-MB-231 cells. The qRT-PCR results further confirmed the deficiency of sGCα1 and sGCβ1 in MDA-MB-231 cells, and partial reduction of sGCβ1 in MCF-7 cells. The expression levels of sGC were lower in breast cancer cells compared to normal breast epithelial cells.

Mujoo *et al*. have shown that ER-negative MDA-MB-468 breast cancer cells express both sGCα1 and sGCβ1 [15]. The sGC in MDA-MB-468 cells is activated properly by NO donor or BAY41-2272, a sGC activator. In contrast, ER-negative SK-Br-3 breast cancer cells express sGCβ1, but not sGCα1. NO donor and BAY41-2272 can hardly activate the sGC in SK-Br-3 cells [15]. We found that the expression of both sGCα1 and sGCβ1 was low or not expressed in ER negative MDA-MB-231 cells. The sGC was poorly activated by SNP in MDA-MB-231 cells. On the other hand, although sGCα1 was significantly expressed in ER positive MCF-7 cells, the expression levels of sGCβ1 were much lower than those of sGCα1. It is possible that the low sGCβ1 level may result in a lower functional dimer between sGCα1 and sGCβ1, which may render MCF-7 cells to respond poorly to SNP treatment. Our results and other groups’ results indicated that the expression of sGC subunits varies with different types of breast cancer cells and is independent of the ER status. The variation of sGC subunits in different breast cancer cells and tumors may reflect the heterogeneity of breast cancer.

DNA methylation that results in gene silencing during tumorigenesis has been observed in Rb, p15, p16, PTEN and ERα [33–35]. Our data showed that Dnmt1, Dnmt3a and/or Dnmt3b levels were elevated in MCF-7 and MDA-MB-231 cells as compared to those in 184A1 cells. We also observed that the promoter region of sGCβ1 in MCF-7 cells and sGCα1 in MDA-MB-231 cells were methylated. 5-aza-dC treatment reduced the promoter methylation of sGCβ1 in MCF-7 cells and of sGCα1 in MDA-MB-231 cells. De-methylation induced the expression of sGCβ1 in MCF-7 cells and both sGCα1 and sGCβ1 in MDA-MB-231 cells. Furthermore, 5-aza-dC treatment allowed MCF-7 and MDA-MB-231 cells to respond to SNP treatment. These results indicated that DNA methylation plays an important role in turning off the expression of sGC subunits in MCF-7 and MDA-MB-231 cells. Interestingly, although sGCβ1 mRNA expression was reduced in MDA-MB-231 cells and its expression was induced by 5-aza-dC treatment, the promoter methylation of sGCβ1 was not observed in these cells, suggesting that methylation may silence the transcription of other factors that regulate sGCβ1 expression in MDA-MB-231 cells.

In the breast tumors isolated from patients, we found that sGCα1 were down-regulated in 6 (No.0002, 0004, 0006, 0007, 2557, 2537) out of 10 patients, while sGCβ1 were down-regulated in 3 (No.0002, 0006, 2557) out of these 6 patients. Patient No.0006 acquired promoter hypermethylation on both sGCα1 and sGCβ1. These results suggested it may be one of the mechanisms regulating mRNA expression of sGC subunits in breast cancer patients. Our results also suggested that promoter hypermethylation of sGCα1 and sGCβ1 may serve as a marker for the early detection of some subsets of breast cancer.

NO and cGMP have been shown to exert either stimulatory or inhibitory effects on cell proliferation and apoptosis [15, 36–42]. The effects of NO and cGMP on growth and cell death are likely cell type- or tissue-specific. In the case of breast cancer cells, the NO-cGMP-PKG pathway has been shown to inhibit the growth and apoptosis of MDA-MB-468 and SK-Br-3 breast cancer cells [15]. We observed that the proliferation of MDA-MB-231 cells is suppressed by over-expression of sGCα1 and sGCβ1 via induction of cell cycle arrest and apoptosis. We also demonstarted that over-expression of sGC reduced tumor incidence, tumor volume, and rate of tumor growth of MDA-MB-231 cells in nude mice. In breast tumor tissues, NO is produced by three isoforms of NO synthase, the nNOS, iNOS, and eNOS [10, 11, 13]. Expression of iNOS and eNOS has been shown to be elevated in breast cancer as compared to that in benign breast epithelium [10, 11, 13]. Down-regulation of sGC in breast tumors may thus protect breast cancer cells from the growth inhibition induced by NO-cGMP and provide a growth advantage.

It would be interesting to investigate whether methylation of sGCα1 and/or sGCβ1 promoters correlates to treatment outcome or overall survival of breast cancer patients. As mRNA levels of sGCα1 and/or sGCβ1 and methylation of sGC promoter can be measured readily, they are potential candidates as diagnostic or prognostic biomarkers for certain subtypes of breast cancer.

## Conclusion

Our study demonstrated that down-regulation of sGC, partially due to promoter methylation, provides growth and survival advantage in human breast cancer cells.
